# Parenchyma-sparing strategy and oncological prognosis in patients with colorectal cancer liver metastases

**DOI:** 10.1186/s12957-022-02579-1

**Published:** 2022-04-17

**Authors:** A. A. Burlaka, D. E. Makhmudov, I. I. Lisnyi, A. V. Paliichuk, V. V. Zvirych, A. V. Lukashenko

**Affiliations:** 1 Abdominal Department of National Cancer Institute, Kyiv, 03022 Ukraine; 2 Colorectal Cancer Department of National Cancer Institute, Kyiv, Ukraine; 3 Department of Anesthesia of National Cancer Institute, Kyiv, Ukraine; 4 Medical Center “Omega-Kyiv”, Kyiv, Ukraine; 5 Science of National Cancer Institute, Kyiv, Ukraine

**Keywords:** Parenchyma-sparing surgery, Colorectal cancer, Bilobar metastases

## Abstract

**Background:**

Preliminary study results demonstrated parenchyma-sparing surgery (PSS) as an effective approach which allowed to remove colorectal cancer (CRC) metastatic lesions within the central liver cites and increased the probability of the liver re-resections.

**Methods:**

The prospective analysis re-evaluation of the 185 CRC patients surgical treatment has been performed.

**Results:**

An overall 5-year survival (OS) of the 185 enrolled patients was 43 ± 7%, and the mean and median value for OS was 48.7 ± 1.9% and 55.2 ± 5 (95% *CI*: 44.4–66.1) months. The 5-year OS for CRC patients whose metastatic lesions were predominantly located within peripheral and central liver segments was 56 ± 8% and 27 ± 9%, respectively (*p* = 0.08). A 5-year disease-free survival (DFS) rates of patients with peripheral and central liver cites metastatic lesions were 31 ± 7 % and 15 ± 7%, *p* = 0,12. And the DFS median was 34.2 and 46.5 months for R_1v_ and R0 cohorts, respectively, *p* = 0.62.

**Conclusions:**

Parenchyma-sparing surgery should be a priority pathway for complex treatment of patients with deeply located lesions of the right liver lobe.

**Trial registration:**

The study is registered in https://www.researchregistry.com/browse-the-registry#home/registrationdetails/5ed9f60863e9bf0016624456/, no. 5679.

## Background

About 1.8 million new cases of colorectal cancer (CRC) are diagnosed worldwide yearly. CRC is ranged 3rd in the structure of the most common malignant neoplasms in Ukraine [1]. Throughout the natural history of CRC, > 50% of patients develop liver metastases [2]. Proper liver resection strategy is the cornerstone of metastatic CRC treatment, because only a successful liver resection significantly improves long-term treatment outcomes (e.g., complete removal of metastatic lesions allows to achieve an overall survival rate of 43–65%) [2]. Most liver resections for CRC demonstrate low postoperative morbidity and mortality rates [3]. However, there is a subcategory of CRC patients with bilobar spread of lesions who require major resections (removal of ≥ 3 anatomical liver segments). The extent of surgery strongly correlates with an increased risk of acute liver failure as well as complications in the early postoperative period [4]. In these cases, treatment success depends on a sufficient amount of future liver remnant ranging from 30 to 40% of total liver volume. Taking into account the indisputable effectiveness of the surgical method, different operation strategies for bilobar forms of CRC have been developed. Artificial stimulation of future liver remnant hypertrophy via the ligation or embolization of the portal vein branches in an attempt to proceed with a two-stage hepatectomy was the most prominent one, while other strategies had been introduced just recently [5]. The main disadvantage of two-stage hepatectomy strategy is the possibility of disease progression after the first surgical stage (“dropout”) [6, 7]. This leads to an inability to complete the entire liver resection, resulting in poor oncological prognosis [8]. In an attempt to overcome this issue, an associating liver partition and portal vein ligation (ALPPS) strategy was introduced [9]. However, ALPPS showed a significant decrease of incomplete liver resections; it was strongly associated with increased postoperative mortality rates (9–15%) [9]. An alternative strategy for bilobar CRC involves parenchyma-sparing surgery (PSS) of all bilobar metastatic lesions within one surgical procedure. Such an approach is considered to be an adapted “cherry-picking technique,” widely used for the subcapsular liver metastases removal [9]. Torzilli et al. expanded indications for PSS by introducing the possibility of simultaneous deep hepatic lesions removal under ultrasound guidance [10]. Such an approach seems capable of reducing the necessity of both portal vein embolization and ligation [11]. Authors have noticed the appropriate skills of intraoperative ultrasound guidance and intraparenchymal skeletization of vessels and Glisonean structures of the 1st and 2nd division from adjacent lesions by R_1_ vascular detachment (R_1v_) approach [12]. However, the results of the PSS in cases of deeply localized lesions (“right venous core,” portal or caval confluences, etc.) remain to be unclear. We have recently published the first results of the PSS study within the central and peripheral liver cites [13]. Preliminary study results demonstrated PSS as an effective approach which allowed to remove metastatic lesions within the central liver cites and increased the probability of the liver re-resections.

The aim of this article was to show the long-term oncological results of PSS strategy for CRC liver surgery.

## Methods

The prospective analysis re-evaluation of the 185 CRC patients surgical treatment has been performed. The description of the liver surgery aspects, study design, inclusion and exclusion criteria, and endpoints has previously been published [14]. Statistical analysis has been performed with Prism 8.0 for MacOS, 8.4.2 version. The normality variables distribution has been checked by the Shapiro–Wilk test. *p* = 0.05 was taken as the critical level of significance when testing statistical hypotheses. Patients’ overall (OS) and disease-free survival (DFS) have been analyzed according to the Kaplan–Meier method. The multivariate Cox regression analysis of factors contributing to overall survival of CRC patients has been performed.

## Results

**Fig. 1:**
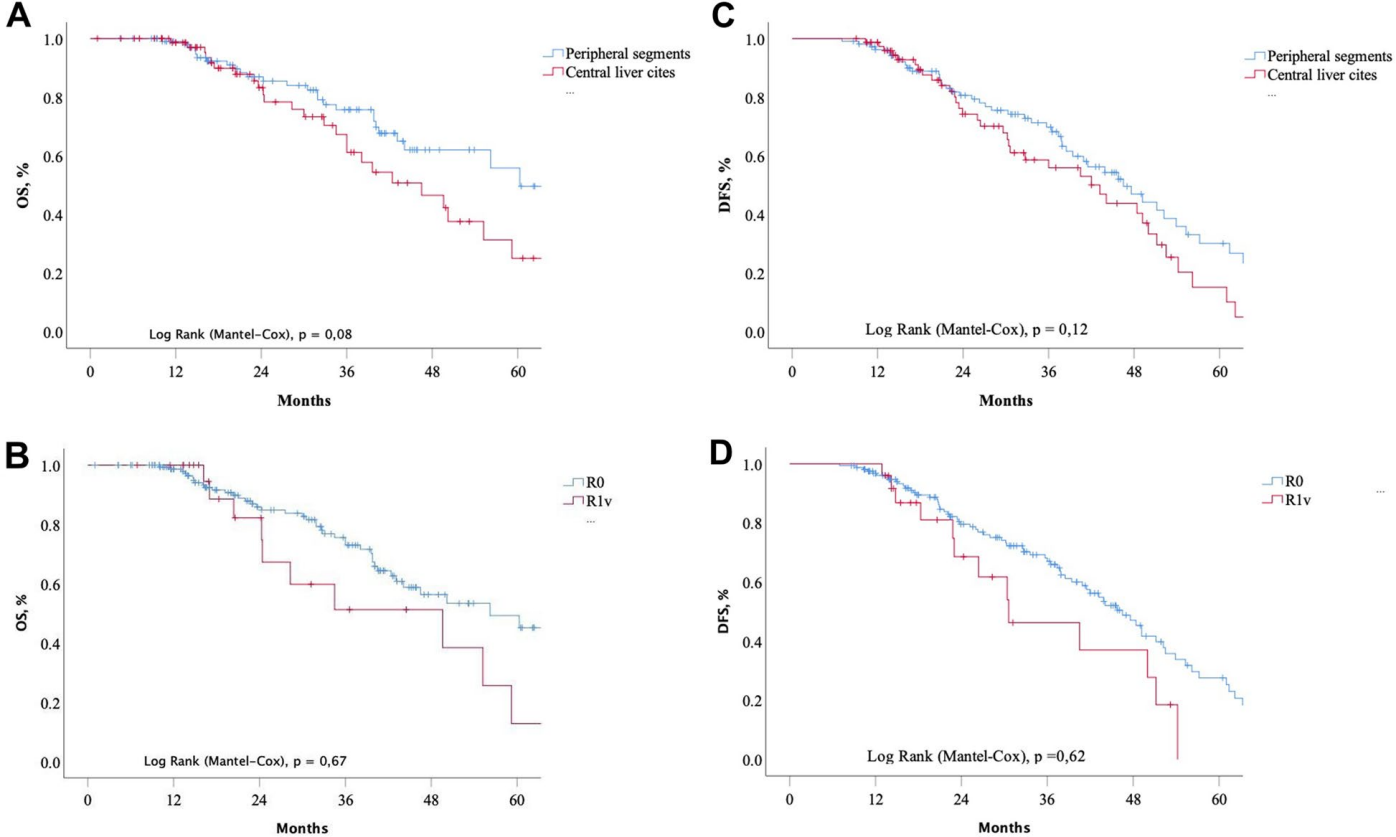
Kaplan–Meier plots: **A** OS of CRC patients with predominant localizations of the metastatic lesions within the peripheral (*n* = 107) and central (*n* = 78) liver segments. **B** OS of CRC patients with liver parenchyma resection margin stratification (R_0_ and R_1_ vascular detachment in 158 and 27 patients, respectively). **С** Disease-free survival of CRC patients with predominant localizations of the metastatic lesions within the peripheral (*n* = 107) and central (*n* = 78) liver segments. **D** Disease-free survival of CRC patients with liver parenchyma resection margin stratification (R_0_ and R_1_ vascular detachment in 158 and 27 patients, respectively)

An overall 5-year survival (OS) of the 185 enrolled patients was 43 ± 7%, and the mean and median value for OS was 48.7 ± 1.9% and 55.2 ± 5 (95% *CI*: 44.4–66.1) months, while CRC patients OS whose metastatic lesions were predominantly located within peripheral and central liver cites was 56 ± 8% and 27 ± 9%, respectively (*p* = 0.08) (Fig. 1A). OS median in the same groups was estimated as 60.3 ± 4.6 (95% *CI*: 51.4–69.3) and 46.5 ± 6.5 (95% *CI*: 33.7–59.3) months, respectively (Fig. 1A).

There were no statistical differences in 5-year OS between cohorts of the patients with R_1v_ and R_0_ resection margin, 48 ± 7% and 13 ± 12 %, respectively, *p* = 0,67 (Fig. 1B). A 5-year DFS of patients with peripheral and central liver cites metastatic lesions was 31 ± 7 % and 15 ± 7%, *p* = 0.12 (Fig. 1C). Whereas cohort (*n* = 27) of CRC patients with R_1v_ margin did not reach a 5-year follow-up, the DFS median was 34.2 and 46.5 months for R_1v_ and R_0_ cohorts, respectively, *p* = 0.62 (Fig. 1D).

In attempt to comprehensively assess the risks of survival and their relation to independent clinical and surgical factors, we performed a comparative analysis of the existing database (see Table 1).

**Table 1:** Univariate and multivariate analysis for overall survival prognosis

	*n*	Univariate		Multivariate	
		Median, months	*P*	B-factor	*P*
**Preoperative factors**					
T_1–2_	27	-	0.32	0.54	0.21
T_3–4_	158	31.6			
N_−_	93	-	0.24	−0.34	0.44
N_+_	78	30.9			
M_0_	82	-	0.21	0.98	0.79
M_1_	103	39.2			
Primary tumor site					
Right colon	28	25.4	0.52	−0.83	0.13
Left colon	157	-			
Lung metastases					
Yes	18	16.7	0.001	−0.41	0.53
No	167	-			
KRAS status				2.3	0.001
Mutation	48	14.6	0.001		
Wild type	137	-			
**Tumor burden of the liver**					
Number of metastatic cites					
1–5	99	39.2	0.67	−0.67	0.12
> 5	86	25.4			
Size of metastatic lesion^a^					
< 50 mm	132	66.2	0.99	0.36	0.45
≥ 50 mm	53	35.1			
Involved segments					
1–3	127	-	0.001	0.96	0.02
≥ 4	58	14.6			
Predominant location of metastatic lesions					
Central	78	-	0.3	0.35	0.46
Peripheral	107	37.4			
**Surgical factors**					
Resection margin					
R_0_	158	-	0.44	−0.66	0.91
R_1v_	27	28.1			

“-”Median survival not reached at the time of analysis; ^a^in case of multiple lesions, the largest lesion size was chosen; *В* Beta coefficient. In current equation, the independent variable is the overall cumulative survival rate

Table 1 contains data from univariate and multivariate analysis (MVA) of risk assessment for overall cumulative survival depending on a number of surgical and clinical factors. According to univariate analysis, the overall survival significantly depended on the presence of lung metastases. MVA variables depended on the presence of KRAS mutation and the involvement of ≥ 4 anatomical liver segments.

## Discussion

The modern concept of CRC is based on the perception of the metastatic process as a chronic illness and not as a lethal disease [15]. That is why we believe that PSS has significant advantages for patients with bilobar metastases of CRC from both pathophysiological and oncological points of view in comparison with two-stage hepatectomies. Our results suggest that adapted PSS strategy was effective for multiple bilobar liver metastases removal regardless of their organ distribution and the number of simultaneously removed lesions. Hence, MVA did not demonstrate any significant impact of excised lesions number on the overall survival. On the contrary, it has been proved that PSS provides perspectives for further successful adjuvant treatment. According to the different sources, about 60% of operated patients have recurrent metastatic liver lesions, while PSS increases the likelihood of re-resection in such patients. Thus, out of 185 operated patients, 1, 2, 3, 4, and 5 liver resections have been performed in 115 (62.2%), 36 (19.4%), 25 (13.5%), 14 (7, 5%), and 1 (0.5%) cases, respectively. We consider this result as satisfactory, given that 1/3 of patients had bilobar metastatic lesions and more than ½ of the patients had recurrent metastatic lesions of the operated parenchyma and successfully underwent re-resection. We believe that a multidisciplinary team should consider several options for systemic anticancer therapy and surgical treatment, including PSS strategy that may be appropriate for most patients with recurrent metastatic disease. Furthermore, the use of nontoxic chemotherapy regimens that will not increase the level of postoperative complications is a fundamentally important factor in the success of the abovementioned treatment. It has been proven that administering more than 12 cycles of chemotherapy can lead to unacceptable levels of postoperative complications and mortality [16]. The survival results from our study refer to worldwide accepted results — the overall 5-year survival rate was 43 ± 7%. We want to emphasize that authors who apply the principles of PSS in treatment of CRC are positive regarding the safety of 1-mm resection margin and R_1v_ resections, which have been mentioned as key components of the entire PSS strategy [17]. This study revealed that the resection margin status (R_0_/R_1v_) had no impact on survival rate in either univariate analysis or MVA. During 5 years, no local recurrence was detected on the radiological follow-ups in the cohort of 27 patients who underwent intraparenchymal preservation of 1–2 order vascular structures. Consequently, we believe that the adaptation of PSS in CRC patients has the potential of liver surgery personification.

## Conclusions

Parenchyma-sparing surgery has shown appropriate surgical and oncological outcomes of treatment of the colorectal cancer patients with bilobar liver metastases. Parenchyma-sparing surgery should be a priority pathway for complex treatment of patients with deeply located lesions of the right liver lobe.

## Data Availability

Not applicable.

## Abbreviations

ALPPS: Associating liver partition and portal vein ligation
CRC: Colorectal cancer
DFS: Disease-free survival
PSS: Parenchyma-sparing surgery
MVA: Multivariate analysis
OS: Overall survival
R_1v_: R_1_ vascular detachment
